# DeepSMCP – Deep-learning powered denoising of Monte Carlo dose distributions within the Swiss Monte Carlo Plan

**DOI:** 10.1016/j.zemedi.2025.02.004

**Published:** 2025-03-17

**Authors:** Hannes A. Loebner, Raphael Joost, Jenny Bertholet, Stavroula Mougiakakou, Michael K. Fix, Peter Manser

**Affiliations:** a Division of Medical Radiation Physics and Department of Radiation Oncology, Inselspital, Bern University Hospital and University of Bern, Bern, Switzerland; b ARTORG Center, University of Bern, Bern, Switzerland

**Keywords:** Monte Carlo, Radiotherapy, Denoising, Deep learning, U-Net, Dose calculation

## Abstract

This work demonstrated the development of a fast, deep-learning framework (DeepSMCP) to mitigate noise in Monte Carlo dose distributions (MC-DDs) of photon treatment plans with high statistical uncertainty (SU) and its integration into the Swiss Monte Carlo Plan (SMCP). To this end, a two-channel input (MC-DD and computed tomography (CT) scan) 3D U-net was trained, validated and tested (80%/10%/10%) on high/low-SU MC-DD-pairs of 106 clinically-motivated VMAT arcs for 29 available CTs, augmented to 3074 pairs. The model was integrated into SMCP to enable a “one-click” workflow of calculating and denoising MC-DDs of high SU to obtain MC-DDs of low SU. The model accuracy was evaluated on the test set using Gamma passing rate (2% global, 2 mm, 10% threshold) comparing denoised and low-SU MC-DD. Calculation time for the whole workflow was recorded. Denoised MC-DDs match low-SU MC-DDs with average (standard deviation) Gamma passing rate of 82.9% (4.7%). Additional application of DeepSMCP to 12 unseen clinically-motivated cases of different treatment sites, including treatment sites not present during training, resulted in an average Gamma passing rate of 91.0%. Denoised DDs were obtained on average in 35.1 s, a 340-fold efficiency gain compared to low-SU MC-DD calculation. DeepSMCP presented a first seamlessly integrated promising denoising framework for MC-DDs.

## 1 Introduction

Monte Carlo (MC) methods are widely considered as the gold-standard for radiation therapy dose calculation owing to their ability to accurately simulate the particle transport in inhomogeneous media to obtain the dose distribution (DD) of radiotherapy plans [1]. However, a large number of particle histories must be simulated to achieve reasonable statistical uncertainty (SU), requiring long calculation times for CPU-based MC dose calculation algorithms [2] and making the use of MC methods in the clinic difficult. GPU-based MC dose calculation for photon [3], [4], [5], [6] and proton/ion-based [7], [8] radiotherapy treatment plans had become increasingly popular due to the substantially reduced calculation time. Nonetheless, in today’s clinical practice, mostly analytical or simplified MC dose calculation algorithms are employed balancing accuracy and calculation time, with calculation time typically in the range of several minutes [9].

Deep-learning (DL) can circumvent the long calculation times with promising accuracy. Usually, a distinction between predicting an optimal achievable dose distribution for a given treatment site and cancer type and dose prediction based on a given set of machine parameters is made. In the first category, U-nets [10], [11], general adversarial networks [12], [13], [14], transformer neural networks [15] and long short-term networks [16] have been used to directly predict a DD based on the CT and/or structure set assuming a specific treatment technique and beam orientation. In the second category, an additional input has been used with similar DL models as in the first category, to guide the dose prediction task. This input ranged from a description of the beam configuration of the treatment plan [17] to cheaply calculated dose distributions, which were then translated into MC dose distributions of low statistical uncertainty [18], [19], [20].

While the first approach substantially speeds up the treatment planning process, it has two main limitations. On the one hand, these models are often dependent on the type and availability of cases used for model training: for instance, a model trained on head and neck cancer [10] is not expected to work for cases in the pelvis region. Moreover, they often depend on the treatment technique. There exist several examples, which only focused on specific setups of intensity modulated radiotherapy (IMRT) [21] or volumetric modulated arc therapy (VMAT) [11]. On the other hand, this approach predicts an optimal DD, which in general differs from the one, which is going to be delivered.

The second approach has the potential to circumvent the problem of case and treatment technique dependence by providing an “informed” input: A promising method is to use MC-DDs of high SU as input and to denoise them using DL. MC-DDs of high SU can be calculated fast, as only a small number of particle histories is simulated. A deep learning network has been employed then used to infer the DD similar to a MC-DD of low SU [19], [20], [22], [23]. This approach also helps decrease the risk of errors, like wrongly estimating doses in regions without any actual doses. Moreover, this approach enables the applicability to new treatment sites and techniques as their task is denoising based on a fast calculated MC-DD with high SU instead of full DD prediction. Combined with a flexible existing MC framework, this approach further allows to circumvent the problem of scarce training data, as random DDs can easily be generated for training.

The Swiss Monte Carlo Plan (SMCP [24], [25]) enables efficient and user-friendly MC dose calculation of radiotherapy treatment plans. It includes a graphical user interface (GUI) and interfaces a commercial treatment planning system (TPS). Beyond dose calculation for state-of-the-art techniques such as intensity modulated radiotherapy (IMRT), or volumetric modulated arc therapy (VMAT), SMCP was used for dose calculation and optimization of research techniques such as dynamic trajectory radiotherapy (DTRT) [26], [27], [28], [29] mixed beam radiotherapy [30], [31], [32]. However, although optimized [33], [34] and parallelizable on multiple CPUs, the MC dose calculations were resource and computation time intensive, especially in the context of MC dose calculation for optimization purposes [32], [35]. In this context, DL methods such as DL powered denoising could provide a substantial efficiency gain.

The goal of this work was therefore to develop a DL powered framework, DeepSMCP, to denoise MC-DD, of VMAT plans and to integrate it into SMCP for a fast, “one-klick” workflow to obtain MC-DD of low SU.

## 2 Materials and Methods

### 2.1 Data sets and model structure

Three different data sets were used in this work (Table 1), each used for a different aim. The first data set was employed to determine the model structure using randomly generated VMAT plans (supplementary material A1). Once the model architecture was determined, the second data set was used to retrain the model (from scratch) with more clinically motivated data. The multi-leaf collimator (MLC) shapes, which defined the beam of the VMAT plans, stemmed from clinically motivated treatment plans. The third data set (application data set) was used to test the models’ applicability and generalizability for clinically motivated dose distributions and treatment sites not present in the second data set. The CTs of the first data set stemmed from multiple CT scanners using different acquisition protocols. For the second and third data set, a Phillips Brilliance CT was used with a kVp of 120 to acquire the CT images. Depending on the tumor location, different slice thicknesses were chosen: 2–3 mm for head and neck, 3 mm for lung, and 3 mm for pelvis. Independent of the data set, CT resolution and slice thickness, an integral conservative gridding algorithm using Hermitian curve interpolation [36] was used to interpolate the CTs to a voxel size of 0.25 × 0.25 × 0.25 cm^3^.

**Table 1:** Description of the data sets used in this work, including their respective objectives and intended applications.

**Data set**	**Description**	**Aim**
1	• CTs of lung, pelvis • Randomly generated treatment plans	To determine the model architecture

2	• CTs of lung, pelvis • Treatment plans with randomly modified MU, isocenter position, collimator rotations, energy	To train the model for clinically relevant beam shapes. (High dose region and tumor location did not necessarily coincide, → to train for “denoising” instead of “tumor location equals high dose region”).

3	• CTs of lung, pelvis, head and neck • Treatment plans	To test the model applicability and generalizability for clinically motivated dose distributions and treatment sites not present in the second data set. (High dose regions and tumor location coincided).

The model structure of DeepSMCP was determined by training and testing five different deep learning models using data set 1. A standard 3D U-net [37] with four layers and skip connections between all encoding and decoding layers was used as main architecture. Skip connections were employed to facilitate the preservation of spatial details during the encoding and decoding process. Convolution kernel sizes are 3 × 3 × 3 voxels. Max-pooling was 3 × 3 × 1 voxels for the first layer and 2 × 2 × 2 voxels in the subsequent layers. The first (irregular) max-pooling was motivated by the coplanar setup of VMAT arcs perpendicular to the z-dimension and the steep dose gradients of VMAT plans in this direction. The input dimension was 192 × 192 voxels in x*y direction and varying size in z-direction. The models were trained to denoise DD with the same number of voxels as the CT. Geometries exceeding 192 voxels in x- and y-direction were cropped centrally and geometries exceeding z voxels in z-direction were handled using a patched-based approach [38]. Resulting overlapping voxels were averaged using TorchIO [39]. Further explanation on overlapping voxels can be found in the supplementary material A2. Geometries with less voxels were padded with zero voxels to fit the model input size.

The five different models differentiated by input size in z-direction, number of input channels (1–2, depending on whether the CT was included as additional input next to the MC-DD of high SU), and batch normalization (Table 2). Details regarding training and evaluation of the five models are described in the supplementary material A1.

**Table 2:** 3D U-net model candidates for DeepSMCP varying by input, input size and batch normalization.

**Model**	**z-size**	**CT**	**Batch normalization**
1	32	yes	no
2	64	yes	no
3	96	yes	no
4	64	no	no
5	64	yes	yes

**Figure 1:**
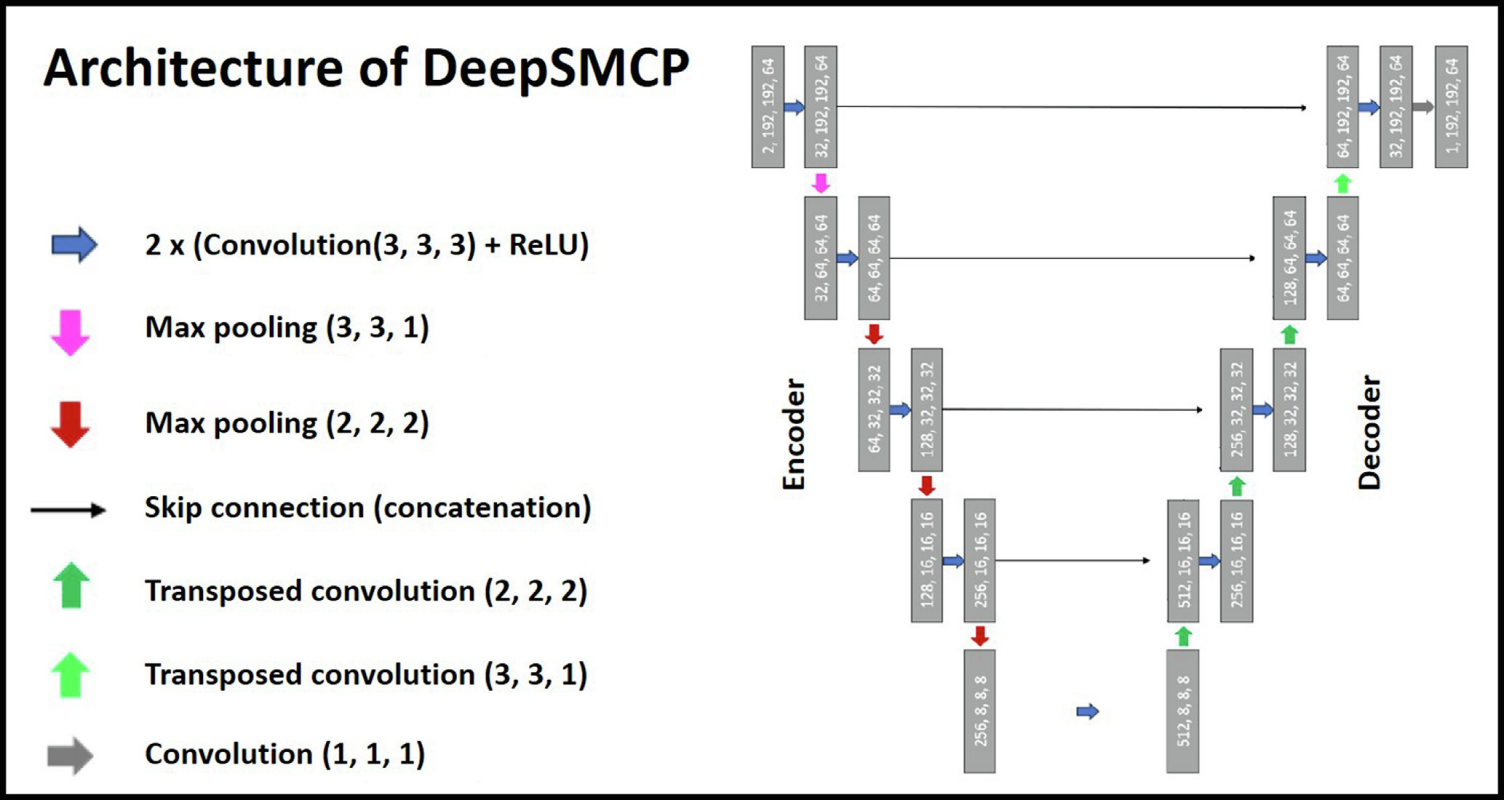
Architecture of DeepSMCP. Architecture of the deep learning model employed in DeepSMCP. It had 2 input channels, one for the high-SU dose distribution and one for the CT.

The best performing model architecture (model 2) in terms of accuracy (supplementary material A1) was selected for DeepSMCP. It had a z-input size of 64, included no batch normalization and had two input channels: one for the DD with high SU and one for the CT, both with size 192 × 192 × 64 voxels (Fig. 1). It had a total of 2.2578*10^7^ parameters.

DeepSMCP was then trained, validated and tested on the second data set consisting of 3074 triplets: a CT and photon MC-DD of high SU as input and a photon MC-DD of low SU as output. To obtain this data set, 106 clinically motivated optimized single VMAT arcs were augmented by a factor 29 to a total of 3074 by randomly changing:

• Monitor units (MU) values of each controlpoints of the arc
• Mean energies (6, 10, 15 MV)
• Isocenter position (±9 cm in x-, ±5 cm in y- and ±10 cm in z-direction, always within the body)
• Collimator rotation

It is important to note, that the MLC shapes of the 106 clinically motivated single VMAT arcs were not modified in order to train with realistic beam shapes. Each of the 106 arcs was assigned to one of 29 available clinically motivated CTs. Subsequently, a “noisy” (1.5*10^6^ particle histories, high SU >60%) and “low-noise” (3*10^8^ particle histories, low SU <2%) MC-DD [40] were calculated using SMCP [25] with VMC++ [41] as dose engine, for each combination of arc and CT. CTs and MC-DDs had the same voxelsize of 0.25 × 0.25 × 0.25 cm^3^. A total of 3074 input-output pairs were obtained. Doses for voxels outside the body structure were deleted.

### 2.2 Training, validation and testing

For training, validation and testing, the data set 2 was divided into training, validation, and test with an approximate 80%/10%/10% split. The split was performed on a CT (case) basis to prevent test leakage. Input and output DD values, as well as the values of the CTs were scaled by a constant factor each to a range approximately between 0 and 1. The constant factors were determined by averaging the normalization factors of each CT and DD of the test set of data set 1.

During training, random patches with size 192 × 192 × 64 were selected from the DDs and CTs. Additionally, random mirroring and/or rotations for multiples of 90° were applied for data-augmentation. The summed-squared-error loss-function and a batch size of two was used. The ADAM optimizer [42] was employed with initial learning rate of 10^−4^. The learning rate decreased by a factor of 5 when the validation loss did not improve for 4 epochs in a row. The training was terminated after the loss function did not improve over the last 30 epochs. The model was trained on a GeForce RTX 3090 GPU.

The model accuracy was evaluated by comparing the low-SU MC-DD (reference) with the denoised DD by means of root-mean-squared error (RMSE) over all voxels and Gamma passing rate on the test set. Gamma evaluation (global) with 2%/2 mm [43] and 3%/3 mm [44] including 10% threshold is denoted as gamma-2 and gamma-3, respectively.

### 2.3 Computation time

**Figure 2:**
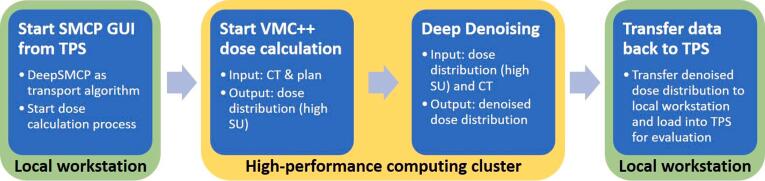
DeepSMCP workflow. Workflow of the dose calculation using DeepSMCP within the Swiss Monte Carlo Plan. The treatment planning system (TPS) and high-performance computing cluster (HPC) were connected using the Eclipse scripting application programming interface.

The computation time on the high-performance computing cluster (Fig. 2) to calculate a high-SU MC-DD, the subsequent pre-processing, denoising and storing was recorded and compared to the calculation time of the low-SU MC-DD.

### 2.4 Integration into SCMP

To enable a fast workflow, DeepSMCP was integrated into SMCP as an additional transport algorithm using a modular approach to enable the interchange of future models within this framework. The general workflow is shown in Fig. 2. First, the SMCP GUI was started from a research version of the Eclipse treatment planning system (Varian, a Siemens Healthineers Company, Erlangen, Germany) as a dose calculation algorithm and DeepSMCP was selected as a transport algorithm. Second, structure set, CT, and plan information were automatically exported to a high-performance computing (HPC) cluster. There, a fast MC dose calculation for 1.5*10^6^ particle histories was performed for each treatment field. Third, this high-SU MC-DD and the CT served as input for denoising. Fourth, the denoised DD was loaded in the Eclipse treatment planning system for plan evaluation.

### 2.5 Application to unseen cases

The framework was applied to 12 unseen clinically motivated cases, data set 3 (4 head and neck, 4 non-small-cell lung, and 4 prostate cases). This data set included a treatment site (head and neck), not present in data set 2, to test the applicability and generalizability of DeepSMCP. DeepSMCP was used to denoise the DD of the whole plan (consisting of multiple (partial) VMAT arcs). The obtained denoised DD were compared to their respective low-SU MC-DDs using RMSE, gamma-2 and gamma-3. The improvement in noise (from high-SU MC-DD to denoised DD) was quantified using the improved signal to noise ratio (ISNR) ‍[23]. Additionally, for the low-SU MC-DD and the denoised DD, dose volume histograms and dose volume parameters were evaluated for the target ($D_{98\%}$ and $D_{2\%}$) and for parallel and serial OARs ($D_{m}$ mean dose, and $D_{2\%}$). Moreover, different volumes of the prescribed dose were compared ($V_{5\%},V_{10\%},V_{20\%},V_{50\%},V_{80\%},V_{90\%}$ and $V_{95\%}$).

## 3 Results

### 3.1 Training

The final model of DeepSMCP was trained for 28 h 19 min and a total of 149 epochs on data set 2. The average mean squared error loss on the training (validation) set exponentially reduced from 615 (828) [a.u.] in epoch 1 to 124.8 (148.5) in epoch 5 to 70.4 (89.4) in epoch 149 and did not fluctuate more than 6.1 (2.2) over the last 20 epochs. The little fluctuation in the loss function across the validation set suggested that the model was not experiencing overfitting to the data set.

### 3.2 Accuracy

On the test set of data set 2, the average RMSE for voxels with more than 10.0% of the maximum dose was 1.19*10^−4^ Gy/MU with a standard deviation of 0.24*10^−4^ Gy/MU. Translated to a representative VMAT arc with 300 MU and a prescription of 50.0 Gy, delivered in 2.0 Gy fractions, this would lead to an average RMSE of 0.9 Gy for the whole treatment. The average gamma-3 and gamma-2 passing rates +/- standard deviation were 94.0 ± 2.3% and 82.9 ± 4.7%, respectively.

### 3.3 Computation time

The average (standard deviation) time to calculate a high-SU MC-DD on one CPU was 32.7 s (7.5 s) evaluated on the test set of data set 2. Subsequent data pre-processing, denoising and storing took on average (standard deviation) 2.6 s (0.3 s). This compared to an average (standard deviation) of 3 h 19 min (42 min) for a low-SU MC dose calculation including storing on one CPU.

### 3.4 Application

**Figure 3:**
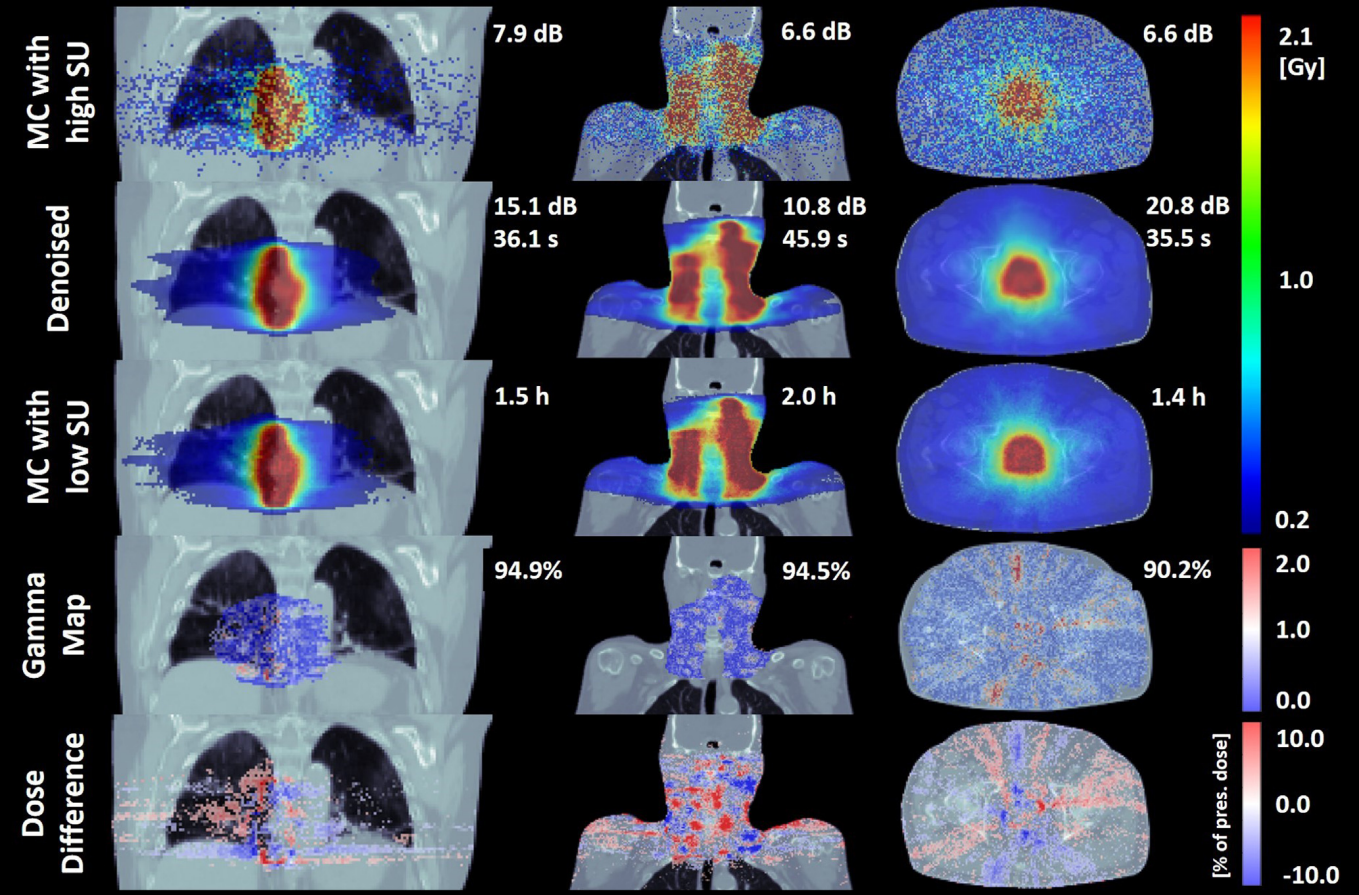
Application of DeepSMCP. DDs with high SU, denoised and low SU are visualized with 10% dose threshold for a lung (left), head and neck (middle) and prostate (right) case, including their computation times and signal to noise ratio as compared to the MC-DD with low SU. Additionally, the gamma map and the dose difference map comparing the denoised DD with low-SU MC-DD are shown. In the gamma map, voxels failing the gamma criteria (2% global/2 mm, 10% threshold) are shown in red tones. The gamma passing rate is shown in the top-right corner of the gamma map. The dose difference is visualized in percent of the prescribed dose.

**Figure 4:**
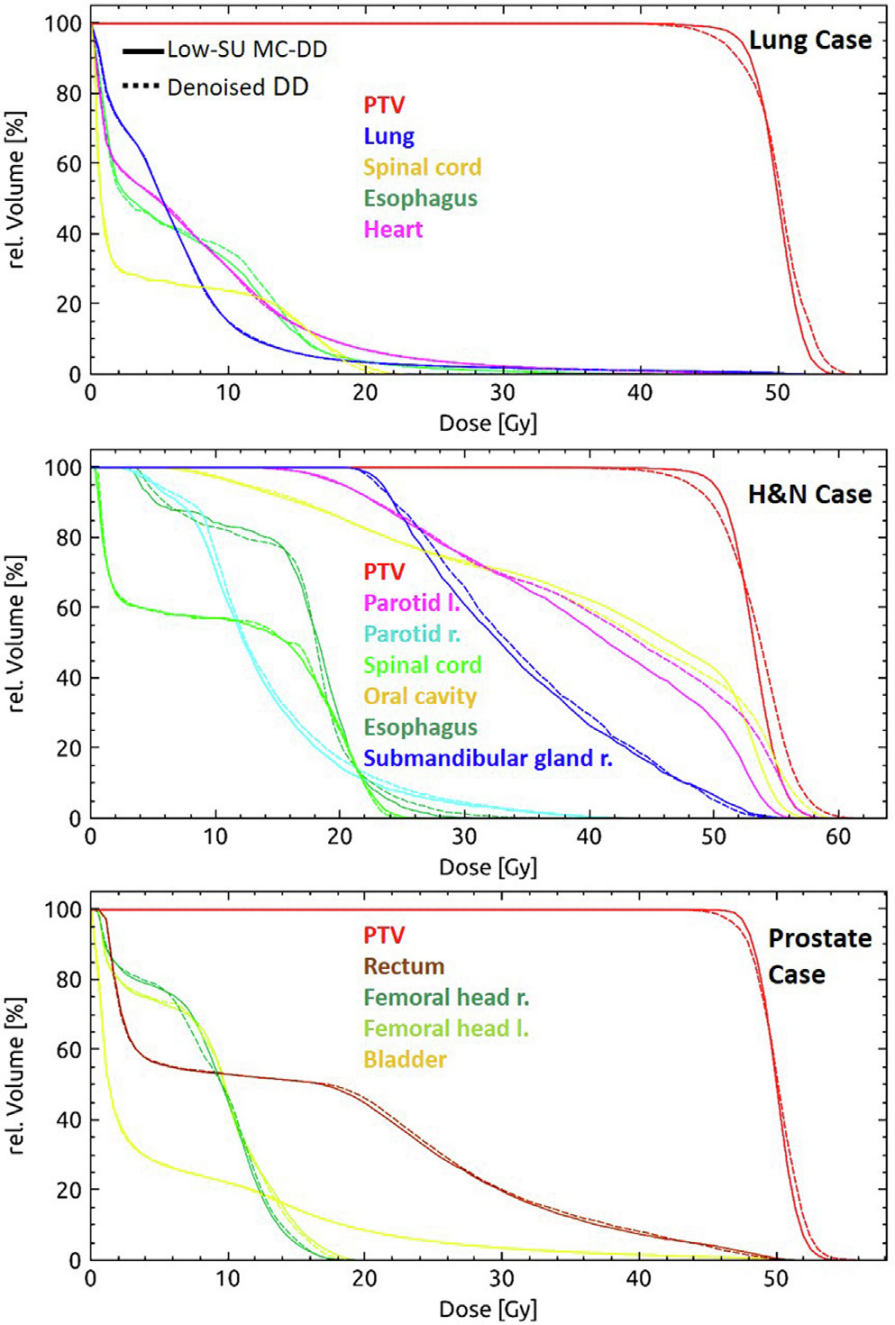
DVHs of three representative cases. DVH for the lung (top), head and neck (H&N, middle) and prostate (bottom) case presented in Figure 3. Denoised and low-SU MC-DD are shown with dashed and solid lines, respectively.

For the 12 unseen cases (data set 3), average [range] RMSE was 1.1*10^−4^ Gy/MU [0.5*10^−4^, 1.7*10^−4^]. Average [range] gamma-3 and gamma-2 were 97.5% [93.4, 98.7] and 91.0% [79.6, 95.9]. An evaluation of the worst case can be found in the supplementary material A3. To evaluate the impact of the baseline noise, the low-SU MC-DD were recomputed 10 times for 5 of the cases. The respective gamma-2 varied by <0.8%. The average [range] ISNR was 9.9 dB [4.2, 15.2]. In Fig. 3, the MC-DD with high SU, denoised and with low SU are shown for three representative cases. The corresponding DVHs are shown in Fig. 4. Greater deviations in DVHs were observed for the target than OARs. Computation time for the denoised DD was < 46 s.

In Table 3, DVH parameters for the target and the closest OARs were evaluated for the 12 cases. Greatest deviations were observed for the target and near max dose quantities.

**Table 3:** Difference in DVH parameters for the target and OARs for the 12 cases for one fraction between the low-SU and denoised DD. The prescription was 2.0 Gy to 50% of the target volume, except for the H&N cases, where it was prescribed to 95% of the target volume.

**Case number**	**Treatment site**	**PTV difference (low-SU MC-DD - denoised DD)**	**OARs difference (low-SU MC-DD - denoised DD)**
1	Lung	$D_{98\%}$ 0.09 Gy (5.3%) $D_{2\%}$ 0.06 Gy (2.8%)	Lung $D_{m}$ -0.01 Gy (2.0%) Heart $D_{2\%}$ 0.03 Gy (2.0%) Esophagus $D_{m}$ 0.0 Gy (0.0%)
2	Lung	$D_{98\%}$ 0.09 Gy (5.3%) $D_{2\%}$ -0.03 Gy (-1.4%)	Heart $D_{2\%}$ 0.0 Gy (0.0%) Esophagus $D_{m}$ 0.0 Gy (0.0%) Lung $D_{m}$ 0.0 Gy (0.0%)
3	Lung	$D_{98\%}$ 0.10 Gy (5.8%) $D_{2\%}$ -0.06 Gy (-2.8%)	Esophagus $D_{m}$ 0.0 Gy (0.0%) Heart $D_{2\%}$ -0.03 Gy (-2.6%) Spinal cord $D_{2\%}$ 0.01 Gy (1.3%) Lung $D_{m}$ 0.0 Gy (0.0%)
4	Lung	$D_{98\%}$ 0.04 Gy (2.3%) $D_{2\%}$ 0.02 Gy (0.9%)	Heart $D_{2\%}$ 0.01 Gy (0.9%) Esophagus $D_{m}$ 0.0 Gy (0.0%) Lung $D_{m}$ 0.0 Gy (0.0%)
5	Pelvis	$D_{98\%}$ 0.07 Gy (3.8%) $D_{2\%}$ 0.08 Gy (3.6%)	Rectum $D_{2\%}$ 0.03 Gy (1.5%) Bladder $D_{2\%}$ -0.01 Gy (-0.6%) Bowel $D_{2\%}$ 0.0 Gy (0.0%)
6	Pelvis	$D_{98\%}$ 0.08 Gy (4.7%) $D_{2\%}$ -0.04 Gy (-1.9%)	Rectum $D_{2\%}$ 0.06 Gy (3.0%) Bladder $D_{2\%}$ 0.0 Gy (0.0%) Bowel $D_{2\%}$ 0.0 Gy (0.0%)
7	Pelvis	$D_{98\%}$ 0.09 Gy (5.4%) $D_{2\%}$ -0.02 Gy (-0.9%)	Rectum $D_{2\%}$ 0.02 Gy (0.9%) Bladder $D_{2\%}$ -0.01 Gy (-0.5%)
8	Pelvis	$D_{98\%}$ 0.02 Gy (1.5%) $D_{2\%}$ -0.06 Gy (-2.8%)	Rectum $D_{2\%}$ 0.01 Gy (0.5%) Bowel $D_{2\%}$ 0.01 Gy (6.5%) Bladder $D_{2\%}$ -0.04 Gy (-1.9%)
9	H&N	$D_{98\%}$ 0.05 Gy (2.9%) $D_{2\%}$ -0.06 Gy (-2.7)	Thyroid $D_{2\%}$ -0.02 Gy (-0.9%) Submand r $D_{m}$ 0.0 Gy (0.0%) Parotid r $D_{m}$ -0.01 Gy (-1.7%)
10	H&N	$D_{98\%}$ 0.10 Gy (5.7%) $D_{2\%}$ 0.01 Gy (0.5%)	Brain $D_{m}$ 0.0 Gy (0.0%) Brainstem $D_{2\%}$ 0.01 Gy (0.7%) Eye r $D_{m}$ 0.0 Gy (0.0%)
11	H&N	$D_{98\%}$ 0.03 Gy (1.5%) $D_{2\%}$ -0.05 Gy (-2.1%)	Submand l $D_{m}$ 0.0 Gy (0.0%) Parotid r $D_{m}$ -0.01 Gy (-1.2%) Brainstem $D_{2\%}$ 0.02 Gy (3.7%)
12	Brain/H&N	$D_{98\%}$ 0.01 Gy (0.6%) $D_{2\%}$ -0.03 Gy (-1.4%)	Brain $D_{m}$ -0.01 Gy (-1.3%) Brain $D_{2\%}$ -0.02 Gy (-1.0%) Brainstem $D_{2\%}$ -0.07 Gy (-5.7%)

$V_{5\%},V_{10\%},V_{20\%},V_{50\%},V_{80\%},V_{90\%}$ and $V_{95\%}$ of the prescribed dose differed on average over the 12 cases by 2.91%, 1.94%, 1.13%, 0.98%, 1.09%, 3.19% and 6.63%, respectively. The maximum difference was observed for $V_{95\%}$ with 10.0% absolute difference between the denoised and the low-SU MC-DD.

## 4 Discussion

We successfully developed a DL powered framework to denoise MC-DDs and integrated it into the SMCP.

The model candidate selection concluded that generally, the different z-input sizes had little impact on gamma-2, gamma-3 and RMSE. Considering the limited memory of the GPU [45], a z-size aligning with the memory of the available GPU should be selected. Additionally, the extension of the target in z-direction should be considered to select an appropriate z-size to minimize patching artefacts in the high-dose region. No substantial accuracy improvement due to the additional CT input was observed. However, in the context of explainable results and mitigating the risk of false dose predictions, the CT input could be beneficial. The inclusion of batch normalization, commonly employed in segmentation tasks and similar U-nets [46], noticeably compromised the accuracy of the denoising process. This could be partly explained by the fact, that batch normalization tended to lose information about absolute dose values. Patching could further magnify this effect, as information from the preceding patch would not be available for subsequent patches. Another reason could be the small batch size (due to memory limit) which compromised the accuracy when using batch normalization. Other normalization techniques, such as group normalization, had been reported to outperform batch normalization for small batch sizes [47]. In future studies, the impact of these normalization techniques on model selection could be investigated.

Denoised DDs with DeepSMCP had an average RMSE of 1.1*10^−‍4^ Gy/MU compared to low-SU MC-DDs, which was in agreement with previous work using a similar approach [23], [44]. Applied to 12 unseen clinically motivated cases, we observed slightly lower gamma passing rates as in previous works which used DL powered denoising or dose prediction methods [20], [23], [44]. However, in this work, the DD of a whole plan was denoised instead of the DD of single beams. Denoising improved the SNR by 9.9 dB on average, a factor 2 smaller than in other works [23]. However, they simulated less/more particle histories for the high/low-SU MC-DD. Nonetheless, visual inspection of our results showed a substantial improvement regarding noise. The DVH-evaluation revealed good agreement (<2% difference) for mean dose quantities between the low-SU and denoised DD. Likewise, the comparison of $V_{X\%}$ of the prescribed dose were on average below 5% difference. However, for near max dose quantities ($D_{2\%})$ and $V_{95\%}$ differences up to 10% were observed. The application of DeepSMCP to denoise DD of clinically motivated treatment plans for treatment sites not present in the training set (i.e., head and neck) showed no substantial differences in terms of accuracy and performance as compared to the other treatment sites. This supported the argument to use DeepSMCP for head and neck treatment plans.

Comparison between the results of the test set of data set 2 and the application to the 12 clinically motivated cases (data set 3), revealed better agreement in terms of RMSE and gamma passing rate between denoised DD and low-SU MC-DD. This could be partly attributed to the loss-function employed during training of DeepSMCP: This loss function penalized larger errors and could in turn lead to too smooth results [48]. The intentional variations in the training data set, particularly in isocenter position and MU, lead to DD including inhomogeneous high dose regions and streak-like features, not commonly observed for VMAT plans in clinical practice. These features were subsequently over-smoothed by DeepSMCP. This coincided with the evaluation of $D_{95\%}$ of the target. The denoised DD had on average a lower $D_{95\%}$, which could be attributed to this over-smoothing and not preserving the steep dose gradient.

The computation time of the denoising was in the same order of magnitude as reported by other works [17], [22], [23]. There exist also models with shorter inference times to obtain denoised DD (e.g., in the ms range) [20]. However, it is important to note, that with our approach, several denoising predictions and patching were performed when the patient geometry exceeds the model geometry. The whole workflow of generating and denoising high-SU MC-DDs was completed within less than a minute, which was faster than most clinically applied dose calculation algorithms [9], [49]. Compared with GPU-based MC dose calculation algorithms, DeepSMCP was on a similar time scale (below 1 min) [6].

The straightforward integration of DeepSMCP into SMCP underlined the flexibility of SMCP. The GUI enabled to directly use DeepSMCP for clinically motivated cases and in a research setting. Potential future applications also include resource-intensive robustness assessments [50] or quality assurance (e.g., as a secondary dose calculation algorithm [51]).

In this work, DeepSMCP had been trained to denoise the DD of VMAT plans. These DD substantially differentiated from the dose distributions of other photon-based treatment techniques such as IMRT plans, which tend to have a more high-dose streaks or DTRT plans, which exploit non-coplanar beam directions. Likewise, the DD of electron-, proton- or mixed beam treatment techniques have unique characteristics differentiating them from VMAT DDs. Although DeepSMCPs task was not to predict a DD from scratch, but to denoise a high-SU MC-DD, we expect that DeepSMCP in its current form will perform worse for these other treatment techniques. However, a strong point of this work was that the training data for other techniques could easily be generated within SMCP. Therefore, DeepSMCP could be extended to different treatment techniques by following the same method as described herein to generate training data for different photon-based treatment techniques [26], [27], [28], [29], intensity modulated electron plans [52], proton therapy plans [53] or mixed beam radiotherapy plans [30], [31], [32].

Moreover, the modular implementation of DeepSMCP within SMCP, permits the seamless integration and interchange of various deep-learning models. This flexibility facilitates the future development and usage of denoising models tailored to specific treatment techniques or sites. Additionally, it enables accuracy and performance comparisons between models within this framework, for instance, to investigate the impact of transfer learning, in future studies.

Even though DeepSMCP produced impressive and promising results for the clinically motivated cases and substantially sped up the dose calculation process, it should be used with caution, especially when considering the accuracy recommendations for dose calculation of ≤2% [54].

## 5 Conclusion

In conclusion, we successfully developed a fast DL powered denoising framework for MC-DDs of VMAT treatment plans (DeepSMCP) and integrated it as a “one-click” solution into SMCP. DeepSMCP showed an efficiency gain of factor 340 and substantial noise reduction with promising accuracy.

## Research data

Research data is not available at this time.

## CRediT authorship contribution statement

**Hannes A. Loebner:** Writing – review & editing, Writing – original draft, Visualization, Software, Methodology, Investigation, Formal analysis, Conceptualization. **Raphael Joost:** Writing – review & editing, Visualization, Software, Methodology, Investigation, Formal analysis, Conceptualization. **Jenny Bertholet:** Writing – review & editing, Methodology, Investigation, Conceptualization. **Stavroula Mougiakakou:** Writing – review & editing, Supervision. **Michael K. Fix:** Writing – review & editing, Supervision, Resources, Project administration, Methodology, Conceptualization. **Peter Manser:** Writing – review & editing, Supervision, Resources, Project administration, Methodology, Conceptualization.

## Declaration of competing interest

The authors declare the following financial interests/personal relationships which may be considered as potential competing interests: This work was supported by Varian, a Siemens Healthineers Company.

JB, MF, PM, declare funding from Grant 200021_185366 of the Swiss National Science Foundation outside of the submitted work.
