# Supplementary material for: DeepSMCP – Deep-learning powered denoising of Monte Carlo dose distributions within the Swiss Monte Carlo Plan
Source: Z Med Phys. 2025 Mar 17;36(1):36–46. doi: 10.1016/j.zemedi.2025.02.004 (PMC12901522; doi:10.1016/j.zemedi.2025.02.004)
Supplement: Supplementary Data 1 [file mmc1.docx]

**Supplementary Material for**

**DeepSMCP – Deep-learning powered denoising of Monte Carlo dose distributions within the Swiss Monte Carlo Plan**

Hannes A. Loebner^a^, Raphael Joost^a^, Jenny Bertholet^a^, Stavroula Mougiakakou^b^, Michael K. Fix^a^, Peter Manser^a^

*^a^Division of Medical Radiation Physics and Department of Radiation Oncology, Inselspital, Bern University Hospital and University of Bern, 3010 Bern, Switzerland*

*^b^ARTORG Center, University of Bern, Murtenstrasse 50, 3008 Bern, Switzerland*

**Supplementary Material A1**

**Model selection**

To determine the most promising model candidate for DeepSMCP the five different models are trained using the same loss function (summed-squared error loss function) and the same optimizer (ADAM) with same initial learning rate (10^-4^) as DeepSMCP. Training concluded when there was no improvement in the loss function over the last 30 epochs.

The dataset for training, validation and testing consisted of 5364 randomly generated volumetric modulated arc treatment (VMAT) plans. These plans involved random multi-leaf collimator shapes for a complete gantry rotation, random assignment of collimator angles, and allocation of monitor unit weights by random sampling between 0 and 1, sorted in ascending order for each control point. Furthermore, a random isocenter was assigned within a range of ± 5 cm in x, y, and z dimensions from the center of the CT. The beam energy was set 6 MV. Subsequently, 12 plans were applied to 447 clinically motivated CTs from open-source datasets, resulting in 5364 pairs of high/low SU MC-DDs. The MC-DDs were computed using SMCP, using 1.5*10^6^ particle histories (high SU >60%) and 3*10^8^ particle histories (low SU <2%), respectively. The dataset was divided into training, validation, and test sets using an 80%/10%/10% split on a case (CT) basis to prevent test leakage. Model accuracy was assessed by comparing the denoised DD against the low SU MC-DD (reference) using root-mean-squared error (RMSE) across all voxels and the Gamma passing with 2%/2 mm (gamma-2) and 3%/3 mm (gamma-3) and a 10% threshold on the test set.

The model accuracy results are displayed in table A1. The best performing model is then selected for DeepSMCP.

*Table A1: Accuracy evaluation of the 5 models including standard variation. The best performing model is marked in bold.*

| **Model** | **RMSE [10^-4^ Gy/MU]** | **Average gamma-2** | **Average gamma-3** |
| --- | --- | --- | --- |
| 1 | 1.21±0.22 | 89.16±3.99 | 97.30±1.69 |
| **2** | **1.16±0.20** | **89.74±4.00** | **97.50±1.66** |
| 3 | 1.15±0.20 | 89.72±3.92 | 97.48±1.61 |
| 4 | 1.16±0.20 | 89.23±4.16 | 97.30±1.78 |
| 5 | 1.26±0.27 | 83.54±4.77 | 94.50±2.55 |

**Supplementary Material A2**

**Patching/Overlap in z-direction**

Following example shall illustrate the patching in z direction for patient geometries exceeding 64 voxels.

Let’s assume a patient with CT length of 185 voxels in z direction. Our model has a z size of 64.

1. Therefore, there will be 185 // 64 = 2 = number of overlapping regions (NOR). That means we need NOR + 1 = 3 predictions to cover the whole patient geometry.

2. To calculate the number of overlapping voxels (NOV) in z direction we obtain: NOV = (3 * 64 - 185) // NOR = 3 with a Rest = (3 * 64 - 185) modulo NOR = 1. The Rest is always appended on the first overlap.

3. In total we have an overlap of 4 voxels in z direction between the first two patches and an overlap of 3 voxels in z direction between the second and third patch.

**Supplementary Material A3**

**Worst case in data set 3**

The worst case in terms of gamma-2 is a prostate case (figure A3), failing gamma passing rate voxels are observed especially in the rectum region, which was filled but also included air pockets. By evaluation of the gamma passing rate, this case can be considered as an outlier with respect to the other eleven investigated cases. The passing rates for the other cases are >89 % for 2% (global) / 2 mm and 10% threshold.


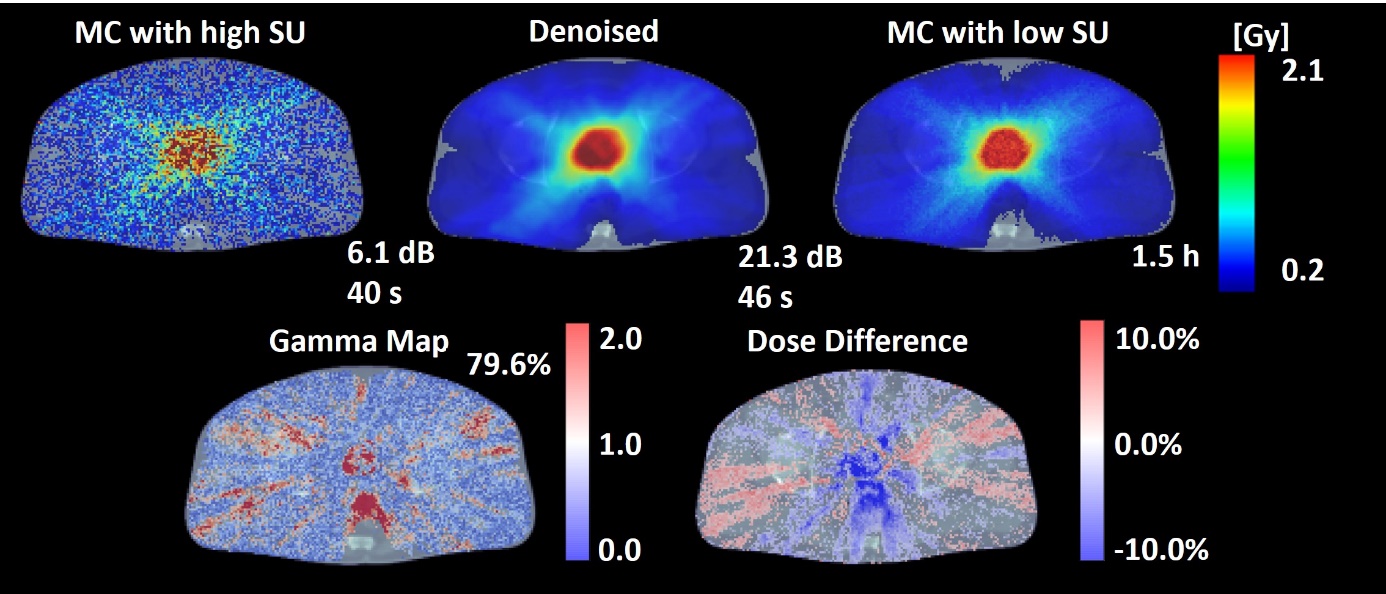


*Figure A3: DDs with high SU, denoised and low SU are visualized with 10% dose threshold, including their computation times and signal to noise ratio as compared to the MC-DD with low SU. Additionally, the gamma map and the dose difference map comparing the denoised DD with low SU DD are shown. In the gamma map, voxels failing the gamma criteria (2% global / 2 mm, 10% threshold) are shown in red tones. The gamma passing rate is shown in the top-right corner of the gamma map. The dose difference is visualized in percent of the prescribed dose.*
